# The Potential Threat of Vertical Transmission in Methicillin-Resistant Staphylococcus Aureus Infection: A Systematic Review 2022

**DOI:** 10.7759/cureus.32366

**Published:** 2022-12-09

**Authors:** Akhil Allakky, Asila A Ferguson, Aujala Irfan Khan, Baraa Abuzainah, Sai Dheeraj Gutlapalli, Dipabali Chaudhuri, Kokab Irfan Khan, Roba Al Shouli, Safeera Khan

**Affiliations:** 1 Internal Medicine, California Institute of Behavioral Neurosciences & Psychology, Fairfield, USA; 2 Psychiatry, California Institute of Behavioral Neurosciences & Psychology, Fairfield, USA; 3 Research, California Institute of Behavioral Neurosciences & Psychology, Fairfield, USA; 4 General Practice, California Institute of Behavioral Neurosciences & Psychology, Fairfield, USA; 5 Pediatrics, California Institute of Behavioral Neurosciences & Psychology, Fairfield, USA

**Keywords:** methicillin-resistant staphylococcus aureus (mrsa), breast milk, neonatal infections, pregnancy, vertical infectious disease transmission, staphylococcus aureus bacteremia

## Abstract

This systematic review paper aimed to assess and analyze the prevalence of maternal colonization of *Staphylococcus aureus* (*S. aureus*) also known as m*ethicillin-sensitive Staphylococcus aureus* (*MSSA*) and m*ethicillin-resistant Staphylococcus aureus* (*MRSA*) in the peripartum period and its significance on vertical transmission to the neonate and if it is a potential threat to the health of newborns. For this, multiple databases, such as PubMed, MEDLINE, ScienceDirect, and the database of Elsevier, were used to scout for relevant articles, and results were reported adhering to the principles set by Preferred Items for Systematic reviews and Meta-Analysis (PRISMA) guidelines 2020. A specific medical subject headings (MeSH) criterion was designed to search for relevant publications on PubMed. A total of 26 articles were finally selected after a meticulous screening process, including detailed inclusion and exclusion criteria, manual reading of titles and abstracts, and availability of accessible full-text articles. A few articles were also selected after going through the citations section of the initially selected papers. Quality appraisal was done on the selected publications.

Maternal colonization of S. aureus is determined to be highly prevalent with the hypothesis that nasal colonization had higher rates than recto-vaginal sites. Increasing maternal age, history of vaginitis, and multiparity were the most common risk factors for *MRSA *and *MSSA* colonization. Premature babies were at the highest risk of *MRSA* colonization. Breast milk is also a risk factor for neonatal MRSA transmission. Through this systematic review, we concluded that although the rate of vertical transmission of MRSA is lower than that of MSSA, we felt that it held significance as neonates with the bug have poor outcomes due to skin and soft tissue infections and there is spread of MRSA to other neonates in the wards and spread to siblings in cases of triplets and quadruplets and even death due to potential MRSA sepsis. Women in Africa and China had high prevalence rates of MRSA and S. aureus which can probably be attributed to a lack of access to adequate healthcare facilities. We recommend screening with regular recto-vaginal swabs and nasal swabs especially in regions with a high burden of *MRSA* to be performed at regular intervals after confirmation of pregnancy, as prevention and screening are effective to avoid serious complications.

## Introduction and background

*Methicillin-resistant Staphylococcus aureus* (*MRSA*) was first observed in 1960 and since then has become notoriously difficult to treat due to the lack of adequate breakthrough drugs [1]. 

Studies have estimated that over 14000 pregnant or postpartum women experience an invasive *MRSA* infection costing about 8.7 billion United States dollars (USD) in the United States of America (USA) alone [2] and around ∼1000 infections and ∼100 deaths occur in children under one year of age [3,4].

Pregnant mothers are susceptible to a variety of infections, and as this study focuses on *staphylococcal* infections, colonization with these bugs is found to be high in prevalence in peripartum mothers due to high estrogen levels and blood glucose levels [5], and *MRSA* infection is a cause of severe diseases in pregnant women [6].

Those in perinatal care are even more likely to be compromised hosts of the bug, in particular premature babies, and those being admitted to the neonatal intensive care unit (NICU); thus, the mother-child relationship in terms of *MRSA* infections has significant importance in the field of obstetrics [6].

Some risk factors associated with neonatal *MRSA* infection are low birth weight, early gestational age at birth, indwelling catheters, duration of antibiotic administration, or the number of days on a ventilator [7].

Though it is widely hypothesized that outbreaks of *MRSA* infections primarily occur in the NICU after contact with colonized mothers or have been a nosocomial infection [8], recent studies have shown that infections have occurred and can occur even without exposure to healthcare settings or healthcare workers [9]. Reported data suggest that infant and maternal colonization of *MRSA* in the USA alone ranges from 1% to 4% which varies across different parts of the country, and it has also been stated that women of African ethnicity are more likely to have higher rates of colonization [4,10].

Although reports on perinatal and vertical transmission of *MRSA* are inadequate, some studies suggest that mother-child transmission of *MRSA* can occur in ways, but not limited to, via colonized genital secretions, placental transmission, mastitis, and also breast milk [11,12].

This systematic review aims to assess the significance and add further information on the vertical transmission of *MRSA* infections in neonates and whether it is a potential threat to the well-being and health of the baby.

Methods

The systematic review was designed, and the results were reported adhering to the principles set by Preferred Items for Systematic reviews and Meta-Analysis (PRISMA) guidelines 2020 [13].

Search Strategy and Data Collection

Databases, such as PubMed, MEDLINE, PubMed Central (PMC), ScienceDirect, and Google scholar, were used to conduct a search using keywords such as neonates, vertical transmission/ mother-child, methicillin resistance, *MRSA*, s*taphylococcal* infections, pregnancy, infectious diseases, and carrier state along with Boolean terms AND, OR. The search was also enhanced using medical subject headings (MeSH) criteria, and appropriate articles pertaining to staphylococcal infections in obstetrics, methicillin resistance, and vertical transmission of *MRSA* in neonates were selected. For other databases, the keywords used were vertical transmission, perinatal *MRSA*, and peripartum *MRSA*.

The MeSH strategy was used as shown in Table 1.

**Table 1:** Data extraction via the MeSH strategy MeSH : Medical subject headings

MeSH strategy used	Results obtained
(( “Staphylococcal Infections/diagnosis”[Majr] OR “Staphylococcal Infections/epidemiology”[Majr] OR “Staphylococcal Infections/microbiology”[Majr] OR “Staphylococcal Infections/transmission”[Majr] )) AND (( “Pregnancy Complications, Infectious/epidemiology”[Majr] OR “Pregnancy Complications, Infectious/microbiology”[Majr] OR “Pregnancy Complications, Infectious/transmission”[Majr] ))	51
(( “Infectious Disease Transmission, Vertical/prevention and control”[Majr] OR “Infectious Disease Transmission, Vertical/statistics and numerical data”[Majr] )) AND ((( “Staphylococcal Infections/diagnosis”[Majr] OR “Staphylococcal Infections/epidemiology”[Majr] OR “Staphylococcal Infections/microbiology”[Majr] OR “Staphylococcal Infections/transmission”[Majr] )) AND (( “Pregnancy Complications, Infectious/epidemiology”[Majr] OR “Pregnancy Complications, Infectious/microbiology”[Majr] OR “Pregnancy Complications, Infectious/transmission”[Majr] )))	2
(( "Carrier State/diagnosis"[Majr] OR "Carrier State/epidemiology"[Majr] OR "Carrier State/microbiology"[Majr] OR "Carrier State/prevention and control"[Majr] OR "Carrier State/statistics and numerical data"[Majr] OR "Carrier State/transmission"[Majr] )) AND ((( "Staphylococcal Infections/diagnosis"[Majr] OR "Staphylococcal Infections/epidemiology"[Majr] OR "Staphylococcal Infections/microbiology"[Majr] OR "Staphylococcal Infections/transmission"[Majr] )) AND (( "Pregnancy Complications, Infectious/epidemiology"[Majr] OR "Pregnancy Complications, Infectious/microbiology"[Majr] OR "Pregnancy Complications, Infectious/transmission"[Majr] )))	8
"Infant, Newborn"[Mesh] AND "Methicillin Resistance"[Majr]	240
("Methicillin Resistance"[Mesh]) AND (( "Infectious Disease Transmission, Vertical/prevention and control"[Majr] OR "Infectious Disease Transmission, Vertical/statistics and numerical data"[Majr] ))	1
( "Pregnancy Complications, Infectious/epidemiology"[Majr] OR "Pregnancy Complications, Infectious/microbiology"[Majr] OR "Pregnancy Complications, Infectious/transmission"[Majr] ) AND - "Methicillin Resistance"[Majr]	10
((( "Infectious Disease Transmission, Vertical/classification"[Mesh] OR "Infectious Disease Transmission, Vertical/economics"[Mesh] OR "Infectious Disease Transmission, Vertical/history"[Mesh] OR "Infectious Disease Transmission, Vertical/prevention and control"[Mesh] OR "Infectious Disease Transmission, Vertical/statistics and numerical data"[Mesh] OR "Infectious Disease Transmission, Vertical/therapy"[Mesh] )) AND (( "Methicillin-Resistant Staphylococcus aureus/analysis"[Majr] OR "Methicillin-Resistant Staphylococcus aureus/drug effects"[Majr] OR "Methicillin-Resistant Staphylococcus aureus/etiology"[Majr] OR "Methicillin-Resistant Staphylococcus aureus/immunology"[Majr] OR "Methicillin-Resistant Staphylococcus aureus/microbiology"[Majr] OR "Methicillin-Resistant Staphylococcus aureus/pathogenicity"[Majr] OR "Methicillin-Resistant Staphylococcus aureus/physiology"[Majr] ))) AND (( "Staphylococcal Infections/diagnosis"[Majr] OR "Staphylococcal Infections/epidemiology"[Majr] OR "Staphylococcal Infections/etiology"[Majr] OR "Staphylococcal Infections/history"[Majr] OR "Staphylococcal Infections/immunology"[Majr] OR "Staphylococcal Infections/microbiology"[Majr] OR "Staphylococcal Infections/pathogenicity"[Majr] OR "Staphylococcal Infections/pathology"[Majr] OR "Staphylococcal Infections/pharmacology"[Majr] OR "Staphylococcal Infections/prevention and control"[Majr] OR "Staphylococcal Infections/statistics and numerical data"[Majr] OR "Staphylococcal Infections/transmission"[Majr] )	1
Total number of titles obtained	313

After obtaining the titles using our MeSH criteria, a manual screening was done by considering a parameter of a timeline of 20 years, and titles not fitting the said criterion were excluded. All the references of the papers were meticulously checked for any potentially overlooked publications. The titles, abstracts, and subject headings were also reviewed for relevance. The outcomes were identified, and data were extracted by the corresponding authors and a stringent peer review was performed as well.

*Inclusion and Exclusion Criteria* 

Our inclusion criteria included papers published in the last 20 years, papers pertaining to the research question, papers published in English, and papers with full text. The population group was also a key factor, and papers that focused on the pregnant female population aged 16-45 years, mothers with active *MRSA* infection in the peripartum period and during delivery, and perinatal *MRSA* infection in neonates were selected.

The excluded papers were those which were papers unrelated to the research topic, published earlier than 20 years ago, papers in other languages, papers focusing on the adult male population, or papers focusing on the geriatric population and papers without full-text access.

Quality Appraisal Tools

A stringent quality check was performed on the extracted publications using the approved quality appraisal tools for each specific type of study. The Newcastle-Ottawa tool was used to assess the quality of observational studies, and the Joanna Briggs Institute checklist was used to assess the quality of clinical case reports and expert opinion articles [14]. The non-bias percentage was calculated in accordance with the respective assessment tool, and studies with a minimum non-bias percentage above 40% were included in the study. The results of the quality appraisal have been tabulated in Table 2.

**Table 2:** Quality appraisal of included studies JBI: Joanna Briggs Institute

Study	Quality assessment tool used	Non-bias percentage
Jimenez-Truque et al., 2012 [4]	Newcastle-Ottawa scale	66.66%
Lin et al., 2018 [5]	Newcastle-Ottawa scale	66.66%
Ogura et al., 2021 [6]	Newcastle-Ottawa scale	88.88%
Holm et al., 2021 [8]	Newcastle-Ottawa scale	66.66%
Parriott et al., 2014 [10]	JBI checklist	100%
Behari et al., 2015 [11]	JBI checklist	87.5%
Morel et al., 2002 [12]	JBI checklist	87.5%
Huang et al., 2009 [15]	Newcastle-Ottawa scale	66.66%
Okiki et al., 2020 [16]	Newcastle-Ottawa scale	44.45%
Chen et al., 2006 [17]	Newcastle-Ottawa scale	66.66%
Creech et al., 2010 [18]	Newcastle-Ottawa scale	66.66%
Chen et al., 2007 [19]	Newcastle-Ottawa scale	88.88%
Gastelum et al., 2005 [20]	JBI checklist	87.5%
Gibbs, 2006 [21]	JBI checklist	100%
Akelere et al., 2013 [22]	Newcastle-Ottawa scale	55.55%
Kriebs, 2008 [23]	JBI checklist	100%
Roca et al., 2017 [24]	Newcastle-Ottawa scale	77.77%
Regev-Yochay et al., 2009 [25]	Newcastle-Ottawa scale	66.6%
Top et al., 2012 [26]	Newcastle-Ottawa scale	77.77%
James et al., 2015 [27]	Newcastle-Ottawa scale	66.66%
Nguyen et al., 2007 [28]	Newcastle-Ottawa scale	66.66%

Results

**Figure 1:**
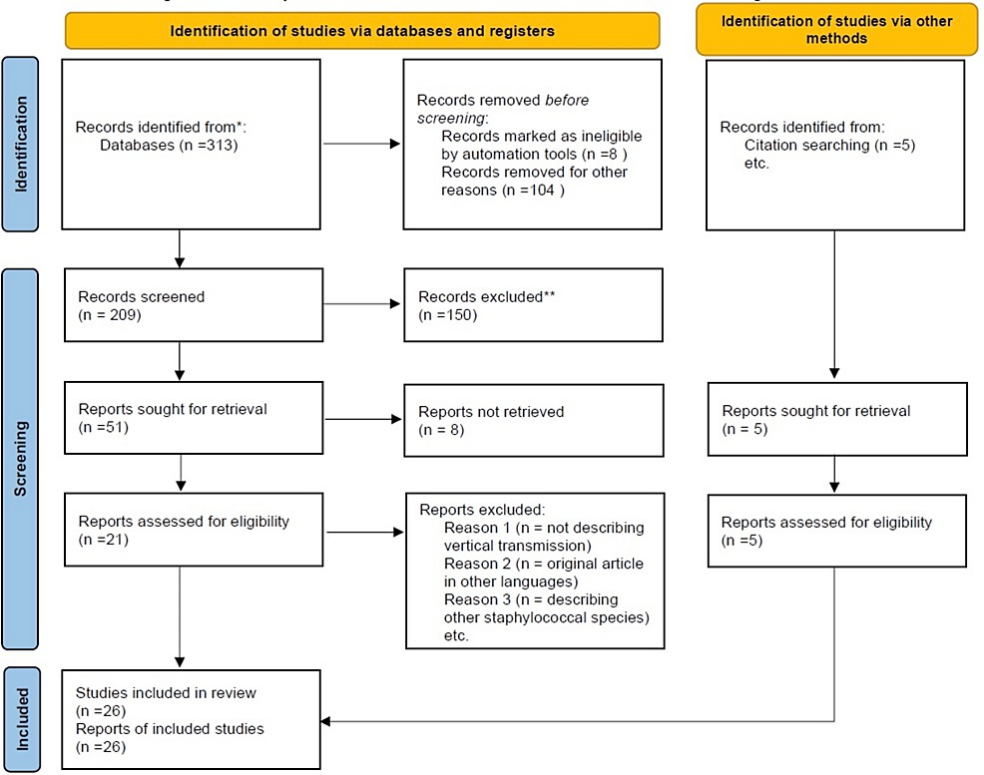
PRISMA flowchart depicting methods and databases used for procuring information * the number of records identified from PubMed and PMC ** the number of records excluded by manual scrutiny of reading the titles and abstracts PRISMA: Preferred Items for Systematic Reviews and Meta-Analysis PMC: PubMed Central

Our primary database search was conducted on PubMed and PMC predominantly using the keywords described earlier. A grand total of 313 publications were found and on adding a time filter of articles within the last 20 years, there was a total of 209 records. Eight papers were repeated and eliminated, resulting in 201 publications that were transferred to the EndNote citation manager (EndNote, Clarivate, London, United Kingdom). Of these 150 were deleted initially, based on manual scrutiny of reading the titles and abstracts as they did not fully pertain to our research topic. Eight more were removed due to a lack of abstracts, full texts, and publications in other foreign languages. Of the remaining 51 papers that described *staphylococcal *infections, 21 were finalized on a meticulous reading of the full text of the paper. An additional five publications were found under the citations section while reading the above 21 papers. Thus, a total of 26 publications were shortlisted for a quality assessment review. A simplified visual representation of the scrutiny process is depicted by the flow chart in Figure 1.

## Review

*S. aureus* or also known as *methicillin-sensitive Staphylococcus aureus (MSSA) *is quite an infamous organism in the field of infectious diseases known for causing wound infections, toxic shock syndrome, and abscesses. In 1959, methicillin was introduced to combat the emerging penicillin-resistant forms of *S. aureus*. But within two years in the United Kingdom, in 1961, there were new reports on the organism acquiring methicillin resistance as well, and thus, the form we know today as *methicillin-resistant Staphylococcus aureus* was discovered. Soon within the next few years, *MRSA* isolates were recovered in other countries in Europe, Asia, and the USA. *MRSA* isolates since then are being increasingly recovered from health centers, nursing homes, and hospitals. A methicillin-resistant penicillin-binding protein is encoded by a methicillin-resistant gene (mecA). mecA is present on a genetic element known as the *staphylococcal* cassette chromosome mec (Sccmec). There are four types of Sccmec, I-IV, which differ in genetic composition and size [12]. *MRSA* infections can be further classified into community-associated *MRSA* (*CA-MRSA*) and hospital-associated *MRSA* (*HA-MRSA*). *HA-MRSA* is associated with type I, II, and III SccmecA while *CA-MRSA* is associated with type IV and type V [15]. It may be noted that for all intents and purposes, this study has used the acronyms *S. aureus* and *MSSA* synonymously and *MRSA* indicates *methicillin-resistant Staphylococcus aureus*.

S. aureus and MRSA Infections in Obstetric Mothers

Several obstetric infections that occur in the obstetric population, such as *toxoplasmosis*, *rubella* *cytomegalovirus*, *herpes simplex*, and *HIV* (*TORCH*) infections, *HIV*, and *hepatitis*, have been described to significantly impact the newborn. This systematic review seeks to shed light on *staphylococcal* infections, particularly *MRSA *among obstetric mothers in the peripartum period and its impact on the health of the neonate.

An observational study conducted in Nigeria in 2020 studied 350 apparently healthy mothers between the ages of 19-43 years attending their antenatal clinic [16]. One hundred and thirty-five women from this study population were swabbed for high vaginal swabs and showed various forms of *staphylococcus *of which 56.1% of the samples were *MSSA*. The *staphylococcal* isolates were found to be least susceptible to penicillin drugs, particularly, amoxicillin/clavulanate- 10.7%, amoxicillin/cloxacillin - 0%, and cloxacillin 0%. This study further reported an *MRSA* prevalence of 14.3% in the study group. It also concluded that *staphylococcal *infections are more prevalent with increasing age (p=0.014) with women above the age of 40 years yielding *staphylococcal* isolation 100% of the time. The limitations of this study were that this study could not find a significant association between *staphylococcal* infections and gestation.

However, a prospective cohort study by Lin et al. (n= 2172) reported that pregnancy is associated with increased carriage of *S. aureus* and *MRSA* in vaginal sites in pregnant women compared to non-pregnant women [5]. The biochemical and hormonal variations in pregnancy, i.e., increased blood glucose levels and higher estrogen levels are hypothesized to be responsible for the increased susceptibility. This study was conducted in two hospitals in Shenzhen, China with apparently healthy pregnant mothers in the gestation period of 35-40 weeks. Among the 2172 women, 338 women had to be apparently excluded due to a lack of adequate medical records. Isolation of the specimen was done by swabbing the vaginal sites as well as the nasal canal which was not done in the previously mentioned Nigerian study. This holds significance as the reported prevalence numbers were higher in nasal sites than vaginal sites. The nasal site isolates for *S. aureus *were 25.8% (473/1834) and for *MRSA* 5.7% (104/1834). The vaginal site samples showed *MSSA* in 7.3% of the population and *MRSA* was isolated from 1.7% of the population. The combined prevalence of *MSSA* and *MRSA* together in a mother was found to be 3.3% and 0.5% for nasal and vaginal sites, respectively. Based on these data, we were able to infer that nasal canal colonization seems to be more significant than vaginal colonization. The overall prevalence of *MRSA* carriage in vaginal sites of pregnant women is 1.7% which is lower than that in the previously mentioned African study. Similarly, we compared the prevalence of *staphylococcal* isolates recovered in patients of the African study with a history of vaginitis/ discharge, 66/146, i.e., 45.2% (p= 0.05) and the Chinese study reported 4/150, i.e., 2.7% (p= 0.81). A cross-sectional study conducted in Copenhagen, Denmark in 2021 (n=1778) reported quite contrasting results compared to the above Chinese and African reports [8]. Only two women, i.e., 0.11% were found to harbor *MRSA*. Both women had been apparently healthy and had no risk factors and the isolation site was the nasal canal, and no isolate was recovered from the recto-vaginal site. This finding made us hypothesize that the quality of healthcare, public hygiene, and strict policies to avoid drug resistance is key. But upon reading a Taiwanese study published in 2009 in The Pediatric Infectious Disease Journal, by Huang et al. we were surprised to note that among the sample size of 499 mothers, only 24 women had *MRSA* colonization (4.8%), of which 20 were isolates from the nasal canal and only three from vaginal sites and one sample from both [15]. 

Though this section in our systematic review primarily speaks about *S. aureus* and *MRSA* infections, we came across a frequently cited study by Chen et al. in various studies that found an association between *Group B streptococcus* (*GBS*) and *S. aureus* colonization [17]. The researchers of this New York-based study reported 743/2963 cultures positive for *GBS* and 507/2963 cultures positive for *S. aureus*, either in combination or alone. They further reported a prevalence odds ratio of 2.1 (95% CI 1.7-2.5, p=<0.001) to *GBS* being significantly associated with *S. aureus* colonization. Of the 507 isolates of S. aureus, 14 were *MRSA,* i.e., 2.8%. Phenotyping of these 14 strains revealed that 13 isolates were *CA-MRSA*. As the study did not report the past medical history of the patients, we could not correlate the parameters of age, antibiotic use, history of vaginitis to see if these were associated with increased *MRSA* carriage. They also could not conclude the rationale behind the significance of *GBS* and *S. aureus* association in pregnancy. These *MRSA* isolates were recovered from the vaginal canal. The reported prevalence is on par with the numbers reported in the above studies and another Pilot study from 2007, conducted in Cleveland, Ohio and reported an *MRSA* prevalence of 2.1% overall [9]. But a study from 2010 by Creech et al. had the highest *MRSA* prevalence of 10.4% (95% CI 5 6.7% to 14.1%), which is the highest reported percentage of *MRSA* carriage we had found in an American-based study [18]. Another 2007 study published by Chen et al. [19] furthered the information garnered from the previous study [17] by stating that only *S. aureus* is associated with *GBS* carriage, but *MRSA* is not. Though the reason is still unclear, it is something that we felt was worth mentioning. 

Vertical Transmission

The main objective of our study was to evaluate if vertical transmission of *staphylococcal* infections is a significant threat to neonatal health and if so, what the risk factors associated with it are. An earlier reported study by Lin et al. which had *MRSA* carriage in the nasal canal at 5.7% and vaginal canal at 1.7% (for the study population of 1834) showed the neonatal incidence of *MRSA* at 0.8% [5]. But when only the study group which had 133 women with vaginal carriage was considered, the neonatal *MRSA* carriage was 2.3% and *S. aureus* incidence was 15.8%. Among the 1701 women who did not have a *staphylococcal* carriage, their neonates had an *MRSA* carriage rate of 0.7% and an *S. aureus* rate of 2.3%. The authors of the study attributed the risk factors for *MRSA* carriage in neonates to be due to the low frequency of vaginal examinations after hospitalization. The number needed to harm (NNH) for *MRSA* in neonates if the mothers had nasal carriage was 138 and that for the vaginal carriage was 23. If the mothers had both nasal and vaginal carriage, the NNH was 42. It was also found that most *MRSA* isolates found in the mothers and neonates from the nasal canal were *CA-MRSA*, whereas those from the vaginal sites were *HA-MRSA*. But when the statistical analysis was done, it was found that there was no significant difference between maternal and neonatal *MRSA* carriage.

The Taiwanese study showed similar results in the incidence rates of neonatal carriage of *MRSA *[15]. The newborn baby population was 501, of which only three had *MRSA* and 40 had *MSSA*. The mothers of the three babies had previous exposure to a healthcare setting during pregnancy. Two mothers were hospitalized, and one mother underwent a surgical procedure during her pregnancy. Another mother worked in healthcare. Owing to the very few cases reported of *MRSA* in the neonate, the researchers were unable to conclude whether the three cases of neonatal *MRSA* were a result of vertical transmission or not.

We came across a case report from 2002, USA, describing all four quadruplet babies born to a single mother infected with *MRSA* [12]. The infants were born 25 weeks preterm and the babies presented with a form of infectious *MRSA* sequelae within 40 days of life. The mother had two prepartum hospital admissions for evaluation and presented with a rupture of membranes, one week before her delivery. She was given a course of ampicillin and erythromycin antibiotics. The prepartum wards did not report any case of *MRSA* infection and colonization either. Further genetic analysis by molecular typing revealed that the strain in the neonates was the same as in the mother, thus supporting the maternal-child relationship in infections. A theory postulated by the researchers of this case report was that the mother acquired the infection from one infant and passed it on to the others. 

Another case report published in 2005 in The Pediatric Infectious Disease Journal supported our theory of vertical transmission [20]. The four premature infants from a single quadruplet gestation were found to have colonization of *MRSA*. The mother initially presented with sequelae similar to mastitis, but the causative organism was not isolated. She continued to express breastmilk and use a breast pump to extract and store milk for her babies. One of the babies died of sepsis which was concluded to be *MRSA* sepsis and shortly after, the three other babies had *MRSA* recovered from various sites on their bodies. Molecular studies confirmed that the strain and bacterial typing are indistinguishable in both the mother and neonates. We also felt that prematurity and multiple gestations played a high risk in this family's increased susceptibility to infections.

Another study published in 2012, by Jimenez-Truque et al. also reported results that showed vertical transmission [4]. They found out that maternal colonization of *S. aureus* and *MRSA* was the highest in prevalence at the commensal of the study, and 38.6% of the mothers had *S. aureus* colonization and 16.6% had *MRSA*. The vertical transmission was found to be at 2.5% for *MRSA* and 9.3% for *S. aureus* among newborn infants which in comparison to our other reviewed studies shows a higher number in prevalence rate. Molecular analysis concluded that 74.8% of *MRSA* isolates were attributed to Sccmec IV type. The researchers also stated that maternal vaginal colonization is an important risk factor that increases vertical transmission in neonates by five times.

A Japanese study from 2021 (n=898) concluded that a group of 55 mothers had *MRSA* colonization and delivered babies, of which 12.7% of neonates had *MRSA* isolates recovered from them at birth [6]. From the remainder population of 843 mothers without *MRSA* colonization, only one baby had an *MRSA* isolate recovered, thereby indicating that maternal colonization does play a significant role in the neonatal outcome. It was also concluded that the method of delivery was vital. No neonate born via C-section had *MRSA* infection while all the reported cases with *MRSA *were neonates born via vaginal birth. Twenty cases of skin and soft tissue infections were reported amongst the neonates who had *MRSA* isolated from various sites. This statement should be taken into consideration with the fact that maternal vaginal colonization had worse neonatal outcomes according to this study.

Summarized highlights of the above-stated points in the observational studies have been tabulated and shown in Table 3.

**Table 3:** Highlights of the results of the observational studies discussed above MSSA: Methicillin-sensitive staphylococcus aureus, MRSA: methicillin-resistant staphylococcus aureus, CA-MRSA: community-acquired methicillin-resistant staphylococcus aureus, Sccmec: staphylococcal cassette chromosome mec, and n/a:  not applicable

Study	Maternal *MSSA* carriage and infection rates	Maternal *MRSA* carriage and infection rates	Neonatal *MSSA* rates	Neonatal *MRSA* rates	Remarks/ comments
Relationship between maternal and neonatal *Staphylococcus aureus c*olonization [4]	38.6%	16.6%	9.3%	2.5%	74.8% attributed to SCCmec IV
A prospective cohort study of *Staphylococcus aureus* and methicillin-resistant *Staphylococcus aureus* carriage in neonates: the role of maternal carriage and phenotypic and molecular characteristics [5]	*MSSA* isolates-606/1834 Nasal – 25.8% Vaginal – 7.3%	*MRSA* isolates-135/1834 Nasal- 5.7% Vaginal – 1.7%	3.3%	0.8%	Predominant strains of *MRSA*: Neonates – Non-typable strain 53.3%, Maternal nasal – type IV strain -51.9% Maternal vaginal- type I strain – 35.5%
Vertical transmission of methicillin-resistant *Staphylococcus aureus* at delivery and its clinical impact: an observational, prospective cohort study [6]	n/a	*MRSA* isolates- 55/898 Nasal – 83.6% Vaginal -16.3%	n/a	2.6%	This study speaks about only *MRSA* vertical transmission. *MRSA* caused skin and soft tissue infections in 20 infant cases.
*Staphylococcus aureus* and *MRSA* colonization rates among gravidas admitted to labor and delivery: a pilot study [9]	*MSSA *isolates-21/96 Nasal- 95.4% Vaginal- 4.7%	*MRSA* isolates-2/21 2.1% (isolated from both nasal and vaginal sites of women with prior *MSSA*)	n/a	n/a	The study theorized that 50% of *MRSA* isolates could be *CA-MRSA* strain *MRSA* was associated with *MSSA* and not found in isolation
Association of *Staphylococcus aureus* colonization in parturient mothers and their babies [15]	*MSSA *isolates-134/449 Nasal – 71.6% Vaginal – 18.6% Both vaginal and nasal-9.2%	*MRSA* isolates 24/499 Nasal-83.3% Vaginal-12.5% Both vaginal and nasal- 4%	*MSSA* isolates- 43/499 Nasal-41.8% Umblicus-41.8% Both nasal and umbilicus-16.2%	*MRSA* isolates-3/499 Nasal-33.3% Umbilicus-66.6%	62.5% of *MRSA* isolates belonged to Sccmec type IV
Occurrence of mecA and blaZ genes in methicillin-resistant *Staphylococcus aureus* associated with vaginitis among pregnant women in Ado-Ekiti, Nigeria [16]	56.1% (based on 135 samples with staphylococcal isolates)	14.3%	n/a	n/a	The total study population of is 350 of which 135 subjects had isolates of required significance. This study speaks about *MSSA* and *MRSA* associations with pregnancy only.
Prevalence of methicillin-sensitive and methicillin-resistant *Staphylococcus aureus* in pregnant women [17]	Recto-vaginal-507/2903 (17.1%)	2.76% (based on 507 samples of *staphylococcus aureus*)	n/a	n/a	12/14 *MRSA* strains were SCCmec IV
Frequency of detection of methicillin-resistant *Staphylococcus aureus* from rectovaginal swabs in pregnant women [18]	29/55 (52.72%)	26/55 (47.22%)	n/a	n/a	27% of *MRSA* show SCCmec IV strains

Limitations

This systematic review did not include any clinical trials as we could not find publications about our topic. Due to a lack of access to a few articles owing to the unavailability of full text, we may have missed out on a few valuable papers as those were excluded from our study.

## Conclusions

Through this systematic review, we tried to assess the significance of maternal-fetal transmission of *S. aureus* and *MRSA* infections during the peripartum period and its impact on the newborn child’s health. Advanced maternal age and lack of access to proper healthcare facilities during pregnancy are the leading risk factors for maternal colonization. Preterm births and NICU admissions are the risk factors for neonatal infections.

 All studies describing the vertical transmission of *MRSA* reported rates between 1 and 5% as opposed to *S. aureus*, which is about 10%. Though *MRSA* rates seem low, it could be a potential threat to poor neonatal outcomes and may lead to sepsis, soft tissue infections, and further spread of *MRSA* to other neonates and even death in some cases. As these cases are caused by virtually preventable causes, we believe that it is apt to recommend regular vaginal swabs at staggered intervals after confirmation of pregnancy based on community rates of *MRSA*. This can be effective in avoiding serious complications during outbreaks. Treatment and prevention protocols may also be viewed toward specific strains of *MRSA* such as Sccmec-IV, as these strains attributed to a large number of isolated samples. We also believe that further studies should be conducted to understand if maternal antibodies have a role to play in modifying the infection and carriage risk of *MRSA* and *MSSA*. 
